# 1-Ethyl-1*H*,6*H*-pyrrolo[2,3-*c*]azepine-4,8(5*H*,7*H*)-dione

**DOI:** 10.1107/S1600536809027378

**Published:** 2009-07-18

**Authors:** Dong Dong Li, Gui Hong Tang, Xiang Chao Zeng, Xing Yan Xu, Gang Huang

**Affiliations:** a Department of Chemistry, Jinan University, Guangzhou, Guangdong 510632, People’s Republic of China

## Abstract

The title compound, C_10_H_12_N_2_O_2_, was synthesized by cyclization of 3-(1-ethyl­pyrrole-2-carboxamido)propanoic acid in the presence of polyphospho­ric acid and diphospho­rus pentoxide. In the crystal structure, adjacent mol­ecules are linked by N—H⋯O hydrogen bonds, forming chains extending along the *b* axis.

## Related literature

For pyrroles sourced from marine organisms, see: Liu *et al.* (2005 ▶). For the bioactivity of pyrrole derivatives, see: Banwell *et al.* (2006 ▶); Sosa *et al.* (2002 ▶). For related structures, see: Zeng (2006 ▶); Zeng *et al.* (2005 ▶).
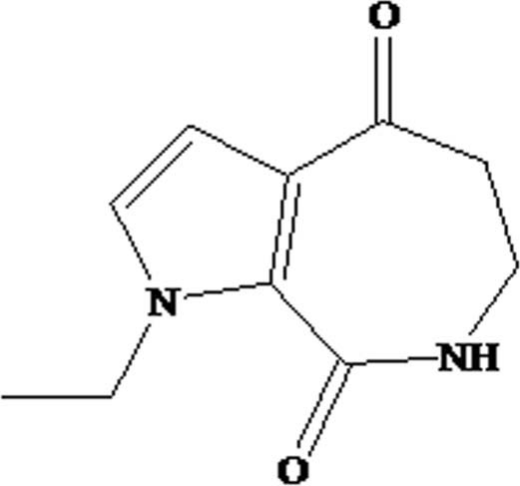


## Experimental

### 

#### Crystal data

C_10_H_12_N_2_O_2_
*M* *_r_* = 192.22
Monoclinic, 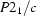

*a* = 11.703 (2) Å
*b* = 7.7863 (13) Å
*c* = 11.0004 (19) Å
β = 113.878 (3)°
*V* = 916.6 (3) Å^3^
*Z* = 4
Mo *K*α radiation
μ = 0.10 mm^−1^
*T* = 173 K
0.46 × 0.45 × 0.30 mm

#### Data collection

Bruker SMART 1K CCD area-detector diffractometer
Absorption correction: multi-scan (*SADABS*; Sheldrick, 1996 ▶) *T*_min_ = 0.956, *T*_max_ = 0.971
4523 measured reflections
1984 independent reflections
1661 reflections with *I* > 2σ(*I*)
*R*_int_ = 0.021

#### Refinement

*R*[*F* ^2^ > 2σ(*F* ^2^)] = 0.037
*wR*(*F* ^2^) = 0.104
*S* = 1.07
1984 reflections
128 parameters
H-atom parameters constrained
Δρ_max_ = 0.28 e Å^−3^
Δρ_min_ = −0.21 e Å^−3^

### 

Data collection: *SMART* (Bruker,1999 ▶); cell refinement: *SAINT-Plus* (Bruker, 1999 ▶); data reduction: *SAINT-Plus*; program(s) used to solve structure: *SHELXS97* (Sheldrick, 2008 ▶); program(s) used to refine structure: *SHELXL97* (Sheldrick, 2008 ▶); molecular graphics: *SHELXTL* (Sheldrick, 2008 ▶); software used to prepare material for publication: *SHELXTL*.

## Supplementary Material

Crystal structure: contains datablocks I, global. DOI: 10.1107/S1600536809027378/jh2089sup1.cif

Structure factors: contains datablocks I. DOI: 10.1107/S1600536809027378/jh2089Isup2.hkl

Additional supplementary materials:  crystallographic information; 3D view; checkCIF report

## Figures and Tables

**Table 1:** Hydrogen-bond geometry (Å, °)

*D*—H⋯*A*	*D*—H	H⋯*A*	*D*⋯*A*	*D*—H⋯*A*
N2—H2*A*⋯O1^i^	0.88	2.12	2.9043 (14)	148

Symmetry code: (i) 
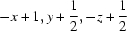
.

**Fig. 1.**
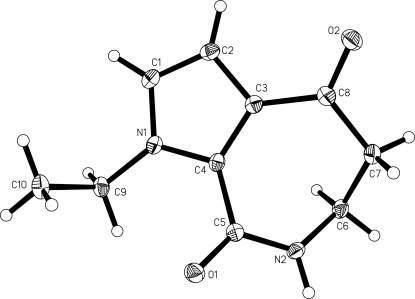
The molecular structure of the title compound, with the atom-numbering scheme. Displacement ellipsoids are drawn at the 30% probability level.

**Fig. 2.**
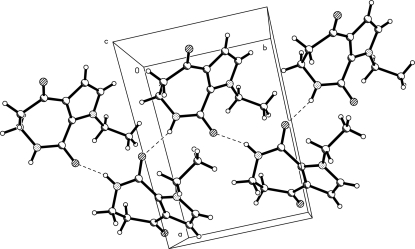
Crystal packing of (I) showing the chain formed by hydrogen bonds (dashed lines).

## supplementary crystallographic information

### Comment

Pyrrole derivatives are well known in many marine organisms (Liu *et al.*, 2005), some show important bioactivities, such as antitumor activity (Banwell *et al.*, 2006) and protein kinase inhibiting activity (Sosa *et al.*, 2002). This is the reason they have attracted our interest. This study is related to our previous structural investigations of 1-Methyl-6,7-dihydropyrrolo[2,3-*c*]azepine-4,8(1*H*,5*H*)-dione (Zeng *et al.*,2005) and 3-bromo-1-methyl-6,7- dihydropyrrolo[2,3-*c*]azepine-4,8(1*H*,5*H*)-dione (Zeng, 2006). In the crystal structure, molecules of the title compound are linked through N2—H2A···O1 hydrogen bonds to form chains extending to the *b* axis (shown in Fig. 2).

### Experimental

3-(1-Ethylpyrrole-2-carboxamido)propanoic acid (0.84 g, 4 mmol) was added to polyphosphoric acid (13 g) to which diphosphorus pentoxide (0.7 g, 5 mmol) had been added, and the mixture magnetically stirred at about 393 K for 0.5 h, and was then poured into ice-water and neutralized with NaOH solution. After filtration, the aqueous solution was extracted four times with ethyl acetate (15 ml). The organic phase was dried with sodium sulfate overnight. The solvent was removed by distillation under reduced pressure, and the pale-yellow solid residue was collected. The crude product was dissolved in the mixture of ethyl acetate (60%) and petroleum ether (40%), colorless monoclinic crystals suitable for X-ray analysis (m.p. 428 K, yield 65.3%) were obtained when the solution was exposed to air at room temperature for 5 d.

### Refinement

All non-H atoms were refined with anisotropic displacement parameters. The H atoms were positioned geometrically [C—H = 0.99Å for CH_2_, 0.98Å for CH_3_, 0.95Å for CH, and N—H = 0.88 Å] and refined using a riding model, with *U*_iso_ = 1.2*U*_eq_ (1.5*U*_eq_ for the methyl group) of the parent atom.

### Crystal data

**Table:**

C_10_H_12_N_2_O_2_	*D*_x_ = 1.393 Mg m^−^^3^
*M**_r_* = 192.22	Melting point: 428 K
Monoclinic, *P*2_1_/*c*	Mo *K*α radiation, λ = 0.71073 Å
*a* = 11.703 (2) Å	Cell parameters from 2801 reflections
*b* = 7.7863 (13) Å	θ = 3.2–27.0°
*c* = 11.0004 (19) Å	µ = 0.10 mm^−^^1^
β = 113.878 (3)°	*T* = 173 K
*V* = 916.6 (3) Å^3^	Block, colourless
*Z* = 4	0.46 × 0.45 × 0.30 mm
*F*(000) = 408	

### Data collection

**Table:**

Bruker SMART 1K CCD area-detector diffractometer	1984 independent reflections
Radiation source: fine-focus sealed tube	1661 reflections with *I* > 2σ(*I*)
graphite	*R*_int_ = 0.021
φ and ω scans	θ_max_ = 27.0°, θ_min_ = 1.9°
Absorption correction: multi-scan (*SADABS*; Sheldrick, 1996)	*h* = −14→10
*T*_min_ = 0.956, *T*_max_ = 0.971	*k* = −9→6
4523 measured reflections	*l* = −11→14

### Refinement

**Table:**

Refinement on *F*^2^	Primary atom site location: structure-invariant direct methods
Least-squares matrix: full	Secondary atom site location: difference Fourier map
*R*[*F*^2^ > 2σ(*F*^2^)] = 0.037	Hydrogen site location: inferred from neighbouring sites
*wR*(*F*^2^) = 0.104	H-atom parameters constrained
*S* = 1.07	*w* = 1/[σ^2^(*F*_o_^2^) + (0.0534*P*)^2^ + 0.2795*P*] where *P* = (*F*_o_^2^ + 2*F*_c_^2^)/3
1984 reflections	(Δ/σ)_max_ = 0.001
128 parameters	Δρ_max_ = 0.28 e Å^−^^3^
0 restraints	Δρ_min_ = −0.21 e Å^−^^3^

### Special details

**Table:**

Geometry. All e.s.d.'s (except the e.s.d. in the dihedral angle between two l.s. planes) are estimated using the full covariance matrix. The cell e.s.d.'s are taken into account individually in the estimation of e.s.d.'s in distances, angles and torsion angles; correlations between e.s.d.'s in cell parameters are only used when they are defined by crystal symmetry. An approximate (isotropic) treatment of cell e.s.d.'s is used for estimating e.s.d.'s involving l.s. planes.
Refinement. Refinement of *F*^2^ against ALL reflections. The weighted *R*-factor *wR* and goodness of fit *S* are based on *F*^2^, conventional *R*-factors *R* are based on *F*, with *F* set to zero for negative *F*^2^. The threshold expression of *F*^2^ > σ(*F*^2^) is used only for calculating *R*-factors(gt) *etc*. and is not relevant to the choice of reflections for refinement. *R*-factors based on *F*^2^ are statistically about twice as large as those based on *F*, and *R*- factors based on ALL data will be even larger.

### Fractional atomic coordinates and isotropic or equivalent isotropic displacement parameters (Å^2^)

**Table:**

	*x*	*y*	*z*	*U*_iso_*/*U*_eq_	
O2	0.07477 (9)	0.05619 (12)	0.30383 (10)	0.0317 (3)	
O1	0.47580 (8)	0.04303 (12)	0.16872 (9)	0.0277 (2)	
N2	0.35948 (10)	0.26744 (13)	0.18305 (11)	0.0239 (3)	
H2A	0.4269	0.3322	0.2129	0.029*	
N1	0.24341 (10)	−0.13996 (13)	0.02340 (10)	0.0222 (3)	
C5	0.37313 (11)	0.10354 (16)	0.15418 (12)	0.0210 (3)	
C1	0.14174 (12)	−0.23379 (16)	0.01642 (13)	0.0255 (3)	
H1	0.1100	−0.3328	−0.0372	0.031*	
C4	0.25889 (11)	−0.00413 (15)	0.10751 (12)	0.0201 (3)	
C7	0.19395 (13)	0.28160 (17)	0.26984 (14)	0.0276 (3)	
H7A	0.2634	0.2860	0.3591	0.033*	
H7B	0.1283	0.3613	0.2705	0.033*	
C3	0.16546 (11)	−0.01426 (15)	0.15559 (12)	0.0212 (3)	
C8	0.14076 (11)	0.10147 (16)	0.24709 (12)	0.0231 (3)	
C2	0.09348 (12)	−0.16271 (16)	0.09844 (13)	0.0247 (3)	
H2	0.0246	−0.2046	0.1144	0.030*	
C9	0.31226 (13)	−0.17678 (16)	−0.05965 (13)	0.0257 (3)	
H9A	0.3682	−0.0790	−0.0542	0.031*	
H9B	0.2521	−0.1882	−0.1534	0.031*	
C10	0.38929 (13)	−0.33927 (18)	−0.01768 (14)	0.0312 (3)	
H10A	0.4517	−0.3265	0.0737	0.047*	
H10B	0.4315	−0.3600	−0.0771	0.047*	
H10C	0.3345	−0.4365	−0.0225	0.047*	
C6	0.24223 (12)	0.34703 (16)	0.16897 (13)	0.0243 (3)	
H6A	0.1785	0.3245	0.0783	0.029*	
H6B	0.2543	0.4729	0.1793	0.029*	

### Atomic displacement parameters (Å^2^)

**Table:**

	*U*^11^	*U*^22^	*U*^33^	*U*^12^	*U*^13^	*U*^23^
O2	0.0380 (6)	0.0290 (5)	0.0366 (5)	0.0024 (4)	0.0237 (5)	0.0032 (4)
O1	0.0232 (5)	0.0257 (5)	0.0347 (5)	0.0027 (4)	0.0123 (4)	0.0015 (4)
N2	0.0223 (5)	0.0189 (5)	0.0302 (6)	−0.0029 (4)	0.0102 (4)	−0.0020 (4)
N1	0.0253 (5)	0.0184 (5)	0.0222 (5)	0.0012 (4)	0.0090 (4)	−0.0004 (4)
C5	0.0232 (6)	0.0204 (6)	0.0199 (6)	0.0015 (5)	0.0091 (5)	0.0027 (5)
C1	0.0263 (7)	0.0192 (6)	0.0271 (6)	−0.0014 (5)	0.0068 (5)	−0.0011 (5)
C4	0.0237 (6)	0.0163 (5)	0.0191 (6)	0.0025 (5)	0.0075 (5)	0.0017 (4)
C7	0.0320 (7)	0.0230 (6)	0.0315 (7)	−0.0004 (5)	0.0168 (6)	−0.0045 (5)
C3	0.0215 (6)	0.0188 (6)	0.0217 (6)	0.0026 (5)	0.0072 (5)	0.0033 (5)
C8	0.0231 (6)	0.0231 (6)	0.0224 (6)	0.0043 (5)	0.0086 (5)	0.0034 (5)
C2	0.0228 (6)	0.0210 (6)	0.0287 (6)	−0.0011 (5)	0.0089 (5)	0.0031 (5)
C9	0.0320 (7)	0.0237 (6)	0.0235 (6)	0.0030 (5)	0.0135 (5)	−0.0007 (5)
C10	0.0341 (8)	0.0283 (7)	0.0308 (7)	0.0077 (6)	0.0128 (6)	−0.0025 (6)
C6	0.0276 (7)	0.0170 (6)	0.0283 (6)	0.0023 (5)	0.0114 (5)	−0.0002 (5)

### Geometric parameters (Å, °)

**Table:**

O2—C8	1.2250 (15)	C7—H7A	0.9900
O1—C5	1.2393 (15)	C7—H7B	0.9900
N2—C5	1.3402 (16)	C3—C2	1.4185 (17)
N2—C6	1.4556 (16)	C3—C8	1.4643 (18)
N2—H2A	0.8800	C2—H2	0.9500
N1—C4	1.3681 (16)	C9—C10	1.5131 (18)
N1—C1	1.3716 (16)	C9—H9A	0.9900
N1—C9	1.4704 (16)	C9—H9B	0.9900
C5—C4	1.4826 (17)	C10—H10A	0.9800
C1—C2	1.3609 (19)	C10—H10B	0.9800
C1—H1	0.9500	C10—H10C	0.9800
C4—C3	1.3964 (17)	C6—H6A	0.9900
C7—C8	1.5137 (18)	C6—H6B	0.9900
C7—C6	1.5223 (18)		
			
C5—N2—C6	125.30 (11)	O2—C8—C3	120.92 (12)
C5—N2—H2A	117.4	O2—C8—C7	118.97 (11)
C6—N2—H2A	117.4	C3—C8—C7	120.09 (11)
C4—N1—C1	108.91 (10)	C1—C2—C3	107.09 (11)
C4—N1—C9	127.96 (11)	C1—C2—H2	126.5
C1—N1—C9	122.85 (11)	C3—C2—H2	126.5
O1—C5—N2	122.26 (12)	N1—C9—C10	112.47 (11)
O1—C5—C4	121.40 (11)	N1—C9—H9A	109.1
N2—C5—C4	116.31 (11)	C10—C9—H9A	109.1
C2—C1—N1	109.25 (11)	N1—C9—H9B	109.1
C2—C1—H1	125.4	C10—C9—H9B	109.1
N1—C1—H1	125.4	H9A—C9—H9B	107.8
N1—C4—C3	107.63 (11)	C9—C10—H10A	109.5
N1—C4—C5	121.69 (11)	C9—C10—H10B	109.5
C3—C4—C5	129.45 (11)	H10A—C10—H10B	109.5
C8—C7—C6	116.02 (11)	C9—C10—H10C	109.5
C8—C7—H7A	108.3	H10A—C10—H10C	109.5
C6—C7—H7A	108.3	H10B—C10—H10C	109.5
C8—C7—H7B	108.3	N2—C6—C7	113.08 (11)
C6—C7—H7B	108.3	N2—C6—H6A	109.0
H7A—C7—H7B	107.4	C7—C6—H6A	109.0
C4—C3—C2	107.08 (11)	N2—C6—H6B	109.0
C4—C3—C8	128.93 (11)	C7—C6—H6B	109.0
C2—C3—C8	123.99 (12)	H6A—C6—H6B	107.8
			
C6—N2—C5—O1	−179.77 (11)	C5—C4—C3—C8	13.5 (2)
C6—N2—C5—C4	−1.61 (17)	C4—C3—C8—O2	−163.02 (12)
C4—N1—C1—C2	−1.80 (14)	C2—C3—C8—O2	17.16 (19)
C9—N1—C1—C2	−176.11 (11)	C4—C3—C8—C7	18.71 (19)
C1—N1—C4—C3	0.68 (13)	C2—C3—C8—C7	−161.10 (12)
C9—N1—C4—C3	174.62 (11)	C6—C7—C8—O2	−162.98 (12)
C1—N1—C4—C5	169.12 (11)	C6—C7—C8—C3	15.32 (17)
C9—N1—C4—C5	−16.93 (18)	N1—C1—C2—C3	2.15 (14)
O1—C5—C4—N1	−30.73 (17)	C4—C3—C2—C1	−1.71 (14)
N2—C5—C4—N1	151.09 (11)	C8—C3—C2—C1	178.14 (11)
O1—C5—C4—C3	134.95 (14)	C4—N1—C9—C10	114.79 (14)
N2—C5—C4—C3	−43.23 (18)	C1—N1—C9—C10	−72.04 (15)
N1—C4—C3—C2	0.63 (13)	C5—N2—C6—C7	69.94 (16)
C5—C4—C3—C2	−166.62 (12)	C8—C7—C6—N2	−72.98 (15)
N1—C4—C3—C8	−179.21 (11)		

### Hydrogen-bond geometry (Å, °)

**Table:**

*D*—H···*A*	*D*—H	H···*A*	*D*···*A*	*D*—H···*A*
N2—H2A···O1^i^	0.88	2.12	2.9043 (14)	148

Symmetry codes: (i) −*x*+1, *y*+1/2, −*z*+1/2.
